# A Prospective Multicenter Analysis of the Mid-term Outcomes of Component Size Variation in Simultaneous Bilateral Total Knee Arthroplasty: Does One-Size Difference Matter?

**DOI:** 10.7759/cureus.94421

**Published:** 2025-10-12

**Authors:** Haresh P Bhalodiya, Rakesh Kumar Singh, Vivek Logani, Vivdh Makwana, IPS Oberoi, Devendra S Solanki, Hemant Wakankar, Avtar K Singh, Buddhadev Chatterjee, Sanjeev Mahajan, Chandrashekhar Yadav, Ashokkumar Thakkar, Udita Chandra, Kunal Aneja, Supreet Bajwa, Ponnanna Machaiah, Ravi Teja Rudraraju, Dolly Singh

**Affiliations:** 1 Orthopaedics, Saviour Hospital, Ahmedabad, IND; 2 Orthopaedics, Global Hospital for Joint Replacement, Kanpur, IND; 3 Joint Replacement and Sports Injury, Paras Hospital, Gurugram, IND; 4 Orthopaedics, Navneet Hi-Tech Hospital, Mumbai, IND; 5 Orthopaedics, Artemis Health Institute, Gurugram, IND; 6 Orthopaedics, Artemis Hospitals, Gurgaon, IND; 7 Orthopaedics, Deenanath Mangeshkar Hospital and Research Center, Pune, IND; 8 Orthopaedics, Amandeep Hospital, Amritsar, IND; 9 Orthopaedics, Apollo Gleneagles Hospitals, Kolkata, IND; 10 Orthopaedics, Fortis Hospital, Ludhiana, IND; 11 Joint Replacement Surgery, All India Institute of Medical Sciences, New Delhi, IND; 12 Clinical Research and Medical Writing, Meril Life Sciences Pvt. Ltd., Vapi, IND; 13 Orthopaedics, Max Super Speciality Hospital, New Delhi, IND; 14 Orthopaedics and Rehabilitation, Naveda Healthcare Centre, New Delhi, IND; 15 Orthopaedics, Wockhardt Hospitals, Mumbai Central, Mumbai, IND; 16 Orthopaedics, Sparsh Hospital, Yeswanthpur, IND; 17 Orthopaedics, Apollo Hospitals, Hyderabad, IND; 18 Clinical Research and Medical Writing, Meril Healthcare Pvt. Ltd., Vapi, IND

**Keywords:** complications, component size difference, patient-reported outcome measures, quality of life, range of motion (rom), simultaneous bilateral total knee arthroplasty

## Abstract

Background

In simultaneous bilateral total knee arthroplasty (bTKA), surgeons often encounter scenarios where femoral or tibial component sizes differ between limbs. While component sizing is routinely individualized, the functional and survivorship implications of such asymmetry remain insufficiently studied. A clearer understanding is clinically relevant, as patient anatomy, bone stock, and soft-tissue balance often necessitate asymmetric sizing, yet evidence guiding its safety and outcomes is limited. This prospective multicenter study evaluated whether inter-limb differences in component sizing influence postoperative function, patient-reported outcomes, or revision risk in bTKA.

Methods

Patients undergoing cemented bTKA with standardized implants (metal femoral components, metal-backed tibial baseplates, and ultra-high-molecular-weight polyethylene inserts) were prospectively enrolled across 10 high-volume centers. Patients were stratified into Subset A (identical component sizes in both knees) and Subset B (different component sizes between knees). Clinical and functional outcomes were assessed preoperatively and postoperatively at regular intervals up to three years. Endpoints included range of motion (ROM), Knee Society Score (KSS), Western Ontario and McMaster Universities Osteoarthritis Index (WOMAC), and 36-Item Short-Form Health Survey (SF-36). Radiographic assessment evaluated alignment, fixation, and implant stability. Minimal clinically important difference (MCID) thresholds were used to confirm the clinical relevance of improvements.

Results

A total of 166 patients (332 knees) were analyzed, with 140 in Subset A and 26 in Subset B. Baseline characteristics were comparable between groups. Both subsets demonstrated significant improvements in ROM and functional scores from baseline to three years (p<0.001 for within-group changes). Subset A achieved higher final ROM (123.6° vs. 118.1°; p<0.001) with less variability, and KSS were also significantly greater in this group (p<0.05).

Subset B, despite inter-knee size differences, showed improvements similar to Subset A in WOMAC scores and demonstrated higher values in selected SF-36 domains, including physical functioning, role limitations, and emotional well-being (p<0.05). No implant-related complications, revisions, or radiographic failures were reported in either group during the three-year follow-up, and radiographs confirmed stable and well-aligned prostheses.

Conclusion

A one-size difference in femoral or tibial components between knees does not compromise clinical or functional outcomes in bTKA. While symmetric sizing may provide slightly greater ROM and KSS improvements, minor asymmetry is not detrimental and may even be associated with enhanced patient-reported quality-of-life domains. These findings support the practice of individualized component selection and reassure surgeons that small inter-limb differences in implant sizing do not negatively impact mid-term function or survivorship.

## Introduction

Total knee arthroplasty (TKA) is a widely accepted and effective intervention for managing end-stage knee osteoarthritis (OA), offering substantial pain relief and restoration of joint function [1]. In cases of bilateral knee involvement, simultaneous bilateral TKA (bTKA) presents a viable option, reducing overall hospitalization, rehabilitation time, and healthcare costs compared to staged procedures [2]. A critical determinant of successful outcomes in bTKA is the precise selection of component sizes for each knee, tailored to individual anatomical variations [3,4].

The sizing of knee implants plays a pivotal role in achieving optimal joint kinematics, stability, and overall functionality post-surgery [5]. The decision to use either the same size or different sizes for femoral and tibial components is multifaceted, involving considerations such as anatomical variations, patient-specific factors, and surgical techniques [5]. This choice has implications for joint biomechanics, ligament balancing, and the overall alignment of the lower extremities, all of which significantly influence the success of bTKA.

Various studies have examined the influence of prosthesis sizing in unilateral TKA cohort studies [6,7]. Incorrect sizing of the femoral component can create a mismatch in the flexion-extension gap [6]. An oversized femoral component may limit flexion space, causing postoperative limitations in flexion and overfilling of the patellofemoral joint [7]. On the other hand, an undersized femoral component can result in instability during flexion. Similarly, a tibial component that is improperly sized can lead to a posterolateral overhang, which might impinge on the popliteus tendon and the posterolateral corner structures [7]. For patients undergoing bTKA, it is essential to size each knee separately, as there can often be variations in knee size within the same patient [2].

Although researchers have examined the correlation between the size of the implant, its alignment, and the balance of soft tissues on the patient's condition after surgery [2], there is a lack of extensive research that explicitly investigates the effects of prostheses of varying sizes in bTKA and the clinical outcomes in the Indian population.

Therefore, this study aims to contribute to the existing body of knowledge by systematically investigating the outcomes of utilizing identical versus different femoral and tibial sizes in patients undergoing bTKA. Through a subset analysis of a prospective cohort study, we intend to assess the impact of implant size selection on postoperative alignment, functional outcomes, and patient satisfaction.

## Materials and methods

Study design and sample size consideration

This study is a subset analysis of the multicenter Freedom 400 study (CTRI/2016/11/007455), which was designed to evaluate implant survivorship and clinical outcomes of the Freedom® Total Knee System in patients undergoing TKA. While the parent study primarily aimed to assess overall survivorship and long-term performance of the implant system, the present subset analyses specifically address questions arising in simultaneous bTKA regarding inter-limb component size variation. In clinical practice, surgeons frequently encounter scenarios where femoral or tibial components of different sizes are required for each knee, yet robust evidence on the safety and functional consequences of such asymmetry remains limited. To address this important knowledge gap, patients who underwent simultaneous bTKA within the Freedom 400 cohort were identified and stratified according to whether both knees received identical component sizes or differed by one size. This subset analysis was therefore undertaken to evaluate whether such size differences influence mid-term functional outcomes, patient-reported measures, or implant survivorship.

Because this is an analysis based on a subset of the Freedom 400 trial, an independent sample size calculation was not applicable. The sample size determination for the main Freedom 400 study was as follows: The main objective of the Freedom 400 will be to demonstrate the survivorship and clinical outcomes of the Freedom® Total Knee System based on the survivorship of the implant. By considering the previous survival rate of 92% from the literature and assuming a survival rate greater than 92%, approximately 364 subjects were required at 95% confidence (α=0.05) with 86% power. With an assumed 10% drop-out rate, the total planned enrollment was increased by 36, resulting in a final target of approximately 400 patients [8].

All 10 participating centers were high-volume joint replacement units distributed across India, each performing more than 100 TKAs annually. Centers were chosen from the Freedom 400 registry network based on consistent reporting quality and adherence to ethical and clinical governance standards. To minimize inter-center variability, a standardized operative and postoperative protocol was implemented across all sites. This included uniform preoperative work-up, anesthesia type, antibiotic prophylaxis, implant system (Freedom® Total Knee System), cementing technique, and rehabilitation protocol. All participating surgeons were fellowship-trained arthroplasty specialists who attended a centralized investigator training program prior to study commencement. The training covered intraoperative sizing and alignment principles, balancing techniques, use of standardized instrumentation, and uniform data recording procedures. Periodic monitoring and audit visits were conducted to ensure continued adherence to the study protocol and data consistency across centers.

Inclusion and exclusion criteria

The criteria for inclusion were patients suffering from primary OA, an advanced end-stage degenerative disorder requiring bTKA. Non-compliance with any of these conditions led to the participant being excluded from the study. The duration of the follow-up period reported in the present study is three years. The comprehensive database comprised patient demographic information, diagnoses, preoperative knee alignment, prosthesis data encompassing component types and sizes, as well as preoperative and postoperative clinical and functional parameters.

Clinical evaluation

Preoperative demographic details, including age, gender, body mass index (BMI), comorbidities, and length of hospitalization, were recorded. Patients underwent baseline assessments and were subsequently evaluated at follow-up intervals of six weeks, six months, one year, and three years. Functional outcomes were assessed using range of motion (ROM) and patient-reported outcome measures (PROMs) [8], which included the Knee Society Score (KSS) [9], the Western Ontario and McMaster Universities Osteoarthritis Index (WOMAC) [10], and the 36-item Short-Form Health Survey (SF-36) [11]. These assessments were conducted by physical therapists who were not directly involved in the surgical procedures. Standardized radiographs were obtained preoperatively, immediately postoperatively, and at each follow-up to evaluate alignment, implant fixation, wear, and evidence of osteolysis.

Statistical analyses

Continuous variables are expressed as mean±standard deviation (SD), and categorical variables are reported as frequencies and percentages. Within-group comparisons over time were conducted using paired t-tests for normally distributed data or the Wilcoxon signed-rank test for non-normal distributions. Between-group comparisons were performed using independent t-tests. Statistical significance was defined as a two-tailed p-value of <0.05. Ninety-five percent confidence intervals (95% CI) were calculated for key continuous outcomes at the final follow-up to quantify estimate precision. Clinically meaningful changes were assessed by comparing outcome improvements against established minimal clinically important difference (MCID) thresholds for each metric. All analyses were conducted using RStudio (R Foundation for Statistical Computing, Vienna, Austria) and IBM SPSS Statistics for Windows, Version 25.0 (IBM Corp., Armonk, New York, United States).

## Results

Baseline details

The mean age of patients in Subset A and Subset B was 64.52±7.49 years and 63.37±5.82 years, respectively. The majority of patients were female in both groups, accounting for 86.43% (n=121) in Subset A and 88.46% (n=23) in Subset B. The average BMI was similar between groups (27.77±4.50 kg/m² in Subset A and 27.99±3.98 kg/m² in Subset B) (Table 1). Comorbidities were also prevalent, with 55.71% (n= 78) in Subset A and 15.38% (n=4) in Subset B having hypertension and 18.57% (n=26) in Subset A and 15.38% (n=4) in Subset B having diabetes mellitus (Table 1).

**Table 1:** Baseline characteristics of patients in study groups (Subset A and Subset B) This table presents the demographic and clinical baseline variables for patients undergoing simultaneous bilateral total knee arthroplasty (bTKA), stratified into Subset A (identical component sizes in both knees) and Subset B (different component sizes between knees). Data are reported as mean±standard deviation for continuous variables and as frequencies with percentages for categorical variables. Between-group comparability was assessed using independent t-tests for continuous variables and chi-squared tests for categorical variables.

Variables	Subset A (same size components) n=140 patients	Subset B (different size components) n=26 patients
Age, years, mean±SD	64.52±7.49	63.37±5.82
Gender, n (%)		
Male	19 (13.57)	3 (11.54)
Female	121 (86.43)	23 (88.46)
Body mass index, kg/m^2^, mean±SD	27.77±4.50	27.99±3.98
Heart rate, bpm, mean±SD	81.00±10.64	79.38±9.28
Systolic blood pressure, mmHg, mean±SD	135.37±19.92	126.69±12.23
Diastolic blood pressure, mmHg, mean±SD	80.89±9.29	78.08±10.41
Primary diagnosis, n (%)		
Osteoarthritis	135 (96.43)	25 (96.15)
Rheumatoid arthritis	3 (2.14)	1 (3.85)
Others	2 (1.43)	-
Advanced degenerative disease of both knees	2 (1.43)	-
Comorbidities, n (%)		
Diabetes mellitus	26 (18.57)	4 (15.38)
Hypertension	78 (55.71)	4 (15.38)

The disposition data highlights a consistent follow-up rate across both subsets over three years, with minimal loss to follow-up (LTF) and withdrawal rates (Table 2). There was a total of two (1.4%) deaths reported in Subset A (one at six months and one at three years) and two (7.7%) in Subset B (at three years) (Table 2).

**Table 2:** Disposition table of patients in study groups (Subset A and Subset B) This table details patient flow during the study, including the number of participants enrolled, lost to follow-up, withdrawn, or deceased at each interval up to three years. The table highlights follow-up adherence in both Subset A and Subset B, demonstrating the completeness of data collection for clinical and functional outcomes.

Category of patients	No. of patients (n=166)	Patient description		
		Subset A (same size components), n (%)	Subset B (different size components), n (%)	
Subjects screened	166	140 (84.3)	26 (15.7)	
Number of subjects enrolled in the study	166	140 (84.3)	26 (15.7)	
Six-week follow-up	Death	1 (0.7)	0	
	Lost to follow-up	0	0	
	Withdrawal	1 (0.7)	0	
Six-month follow-up	Death	1 (0.7)	0	
	Lost to follow-up	0	0	
	Withdrawal	3 (2.1)	0	
One-year follow-up	Death	1 (0.7)		0
	Lost to follow-up	0		0
	Withdrawal	3 (2.1)		0
Three-year follow-up	Death	2 (1.4)		2 (7.7)
	Lost to follow-up	0		0
	Withdrawal	3 (2.1)		0

Component details

Subset A included 140 patients (100%) who received identical femoral and tibial component sizes in both knees, accounting for 280 components in total. In Subset B, 26 patients (100%) received components with a one-size difference between knees, totaling 52 components (Table 3).

**Table 3:** Distribution of femoral, tibial baseplate, and patellar component sizes used in Subsets A and B This table outlines implant characteristics, listing the frequency and percentage of specific femoral, tibial, and patellar component sizes utilized in each subgroup. The distribution highlights the predominance of commonly used sizes and illustrates the occurrence of inter-limb asymmetry (one-size or two-size differences) in Subset B. This contextualizes the rationale for analyzing the effect of component size differences on outcomes.

Components	Subset A, n (%) subjects=140 (n=280 knees)	Subset B, n (%) subjects=26 (n=52 knees)
Femoral component		
Size A	2 (0.71)	2 (3.85)
Size B	86 (30.71)	9 (17.31)
Size C	108 (38.57)	20 (38.46)
Size D	26 (9.29)	14 (26.92)
Size E	42 (15)	5 (9.62)
Size F	16 (5.71)	2 (3.85)
Tibial base plate size		
Size 1	14 (5)	2 (3.85)
Size 2	121 (43.21)	26 (50)
Size 3	79 (28.21)	12 (23.08)
Size 4	28 (10)	9 (17.31)
Size 5	22 (7.86)	3 (5.77)
Size 6	16 (5.71)	0 (0)
Patellar component	Subjects=15 (n=30 knees)	Subject=1 (n=2 knees)
28 mm	0	0
31 mm	14 (43.75)	0
34 mm	16 (50)	2 (100)

Subset A (Same Size Components)

The most frequently used femoral component sizes were C (A/P=58 mm; M/L=62 mm; n=108; 38.57%) and B (A/P=54 mm; M/L=58 mm; n=86; 30.71%) across 280 knees. For tibial baseplates, the most common sizes were Size 2 (A/P=40 mm; M/L=62 mm; n=121; 43.21%) and Size 3 (A/P=42 mm; M/L=66 mm; n=79; 28.21%). Patellar components were used in 15 patients, with 31 mm and 34 mm diameters being the most commonly implanted sizes.

Subset B (Different Size Components)

The most commonly used sizes were C (A/P=58 mm; M/L=62 mm; 38.46%) and D (A/P=60 mm; M/L=65 mm; 26.92%) across 52 knees. For tibial components, 16 patients had identical tibial baseplate sizes in both knees, nine patients had a one-size difference, and one patient (70.3-year-old man) had a two-size difference (right: Size 7; A/P=52 mm; M/L=76 mm; left: Size 5; A/P=48 mm; M/L=71 mm). All patients received 9 mm polyethylene inserts. A 34 mm patellar component was implanted bilaterally in one patient (female, aged 61.6 years).

Implant survivorship and complications

**Figure 1:**
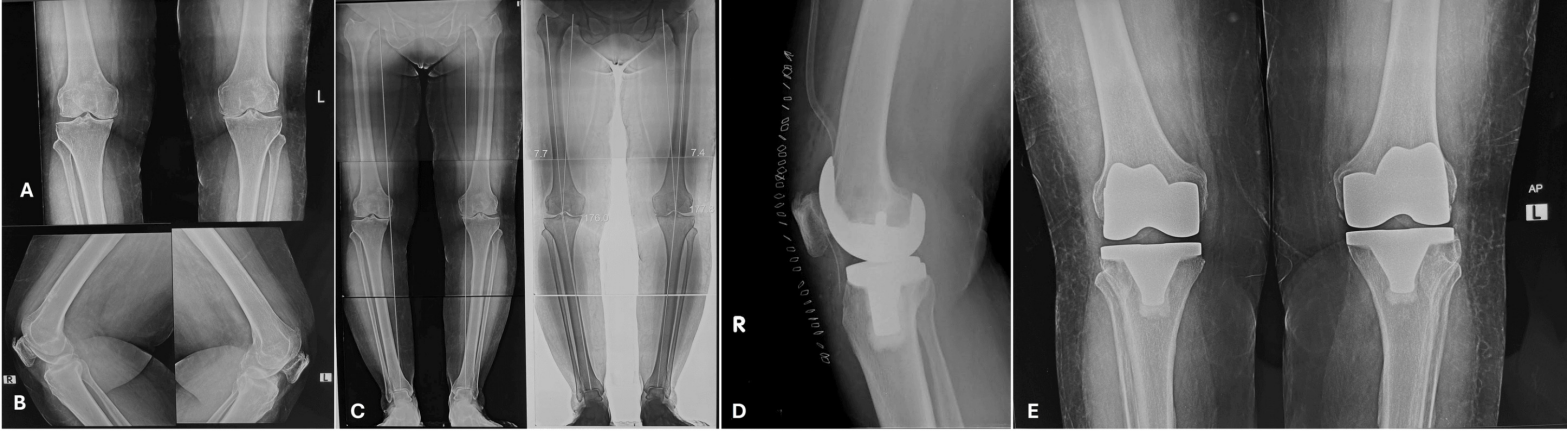
Radiographic images of knees pre- and postoperatively Panel A shows the AP view of the diseased knee. Panel B shows the lateral view. Panel C presents the full-length orthoscanogram depicting the hip-knee-ankle axis for alignment assessment. Panels D and E display postoperative radiographs (lateral and AP views, respectively) demonstrating stable placement of the MBC implant during the three-year study period. These images illustrate the surgical correction of deformity and confirm prosthesis stability and alignment. AP: anteroposterior; MBC: metal-backed cemented

No implant-related revisions or component size-related complications were observed over the three-year follow-up, indicating a 100% implant survival rate. Radiographic evaluations confirmed stable, well-aligned prostheses in all cases (Figure 1).

Four deaths were reported: two in each group. In Subset A, one patient died due to cardiopulmonary arrest secondary to renal failure and acidosis at six weeks postoperatively; another died of pneumonia at three years. In Subset B, one patient died of natural causes and another of a lung infection at the three-year mark. All perioperative and early recoveries were uneventful, and none of the events were attributable to the implant or surgical technique.

One patient in Subset B experienced a mild bilateral skin rash around the knees at six months, which was resolved with medical management. No further symptoms were reported on any follow-up.

These findings affirm the safety and mid-term durability of the metal-based implant system in both symmetric and asymmetric component sizing during simultaneous bTKA.

ROM

Both subsets demonstrated significant improvements in knee ROM from baseline to the three-year follow-up (p<0.001 for both) (Table 4). In Subset A, the mean ROM improved from 94.7°±18.9° at baseline to 123.6°±3.1° at three years, while in Subset B, ROM increased from 96.1°±14.2° to 118.1°±9.6°. Subset A achieved slightly higher final ROM values with narrower variability, suggesting more consistent outcomes. Although the magnitude of improvement (25-30°) was greater than the MCID of 10°, emphasis is placed on the absolute final ROM achieved, which falls within a favorable functional range for daily activities. Importantly, a one-size difference in component selection did not compromise the capacity to regain functional knee motion.

**Table 4:** Comparison of ROM from baseline to three-year follow-up in Subsets A and B This table summarizes improvements in knee ROM for both subgroups over three years. The mean ROM values with standard deviations are shown at baseline and follow-up, alongside the absolute mean difference and 95% CI. Statistical significance was evaluated using paired t-tests for within-group comparisons and independent t-tests for between-group comparisons. The table also reports whether observed changes exceeded the MCID, facilitating the interpretation of clinical relevance. ROM: range of motion; MCID: minimal clinically important difference; FU: follow-up; °: degrees

		Baseline	1 year	3 years	Value (baseline vs. 3-year FU)	MCID threshold	MCID achieved	95% CI
ROM (°)	Subset A (same size components)	94.71±18.90	113.67±9.37	123.62±3.09	<0.001	10	Yes	25.71 to 32.11
	Subset B (different size components)	96.10±14.15	109.88±12.88	118.13±9.64	<0.001	10	Yes	15.11 to 28.95

PROMs

**Figure 2:**
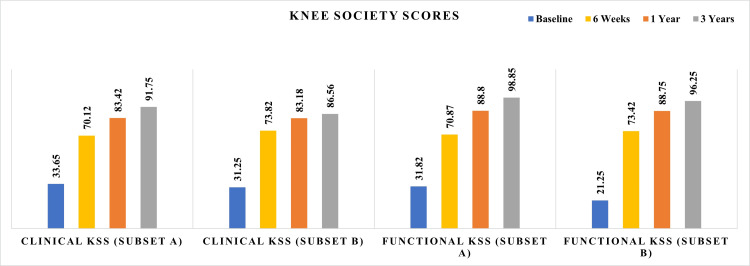
Three-year outcomes of KSS in both study groups (Subsets A and B) Data are presented as mean values with standard deviation/error bars. Statistical comparisons were conducted using independent t-tests at three years, with significance levels indicated. The figure demonstrates that both groups achieved significant functional improvements, with Subset A showing marginally higher final scores. KSS: Knee Society Score

Both Subset A and Subset B exhibited substantial and statistically significant improvements in both clinical and functional KSS over the three-year follow-up (p<0.001 for within-group comparisons). In Subset A, the clinical KSS improved from 33.65±15.81 at baseline to 91.75±7.12 at three years (95% CI: 90.38-93.12), and the functional KSS rose from 31.82±19.30 to 98.85±3.57 (95% CI: 98.16-99.54). In Subset B, the clinical KSS improved from 31.25±15.97 to 86.56±14.25 (95% CI: 79.58-93.54), and the functional KSS from 21.25±27.93 to 96.25±6.19 (95% CI: 93.22-99.28) (Figure 2).

Both groups exceeded the MCID threshold of 8 points for KSS domains, confirming that the improvements were clinically meaningful. Notably, statistical comparison revealed significantly higher three-year KSS scores in Subset A compared to Subset B for both the clinical domain (p=0.0227) and functional domain (p=0.0170), suggesting that same size components may offer slightly more consistent knee-specific outcomes.

Nevertheless, Subset B also achieved excellent functional scores with only modestly greater variability, reinforcing that implant size asymmetry does not compromise the recovery trajectory or patient function at mid-term follow-up.

**Figure 3:**
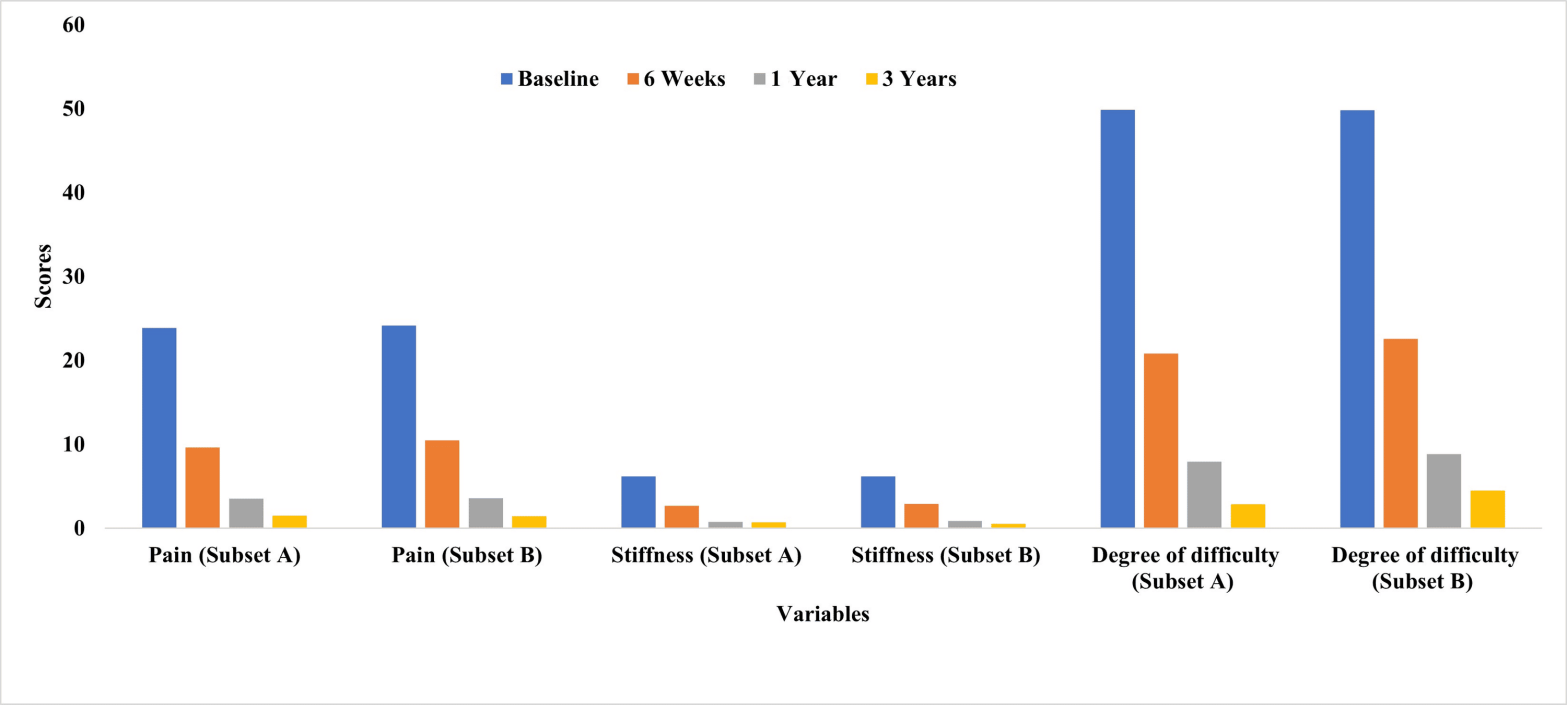
Three-year outcomes of WOMAC scores in both study groups (Subsets A and B) WOMAC: Western Ontario and McMaster Universities Osteoarthritis Index

For WOMAC scores, both Subset A and Subset B demonstrated significant and clinically meaningful improvements across all subdomains, namely, pain, stiffness, and functional difficulty, over the three-year follow-up (p<0.001 for within-group comparisons). In Subset A, the mean pain scores improved from 23.86±5.84 at baseline to 1.47±2.00 at three years (95% CI: 1.23-1.71), while Subset B improved from 24.15±4.71 to 1.43±1.90 (95% CI: 0.88-1.98). Stiffness scores improved to 0.70±0.72 in Subset A and 0.54±0.81 in Subset B, both exceeding the MCID threshold of 2 points. Similarly, the degree of difficulty dropped from 49.89±12.12 to 2.86±4.69 in Subset A and from 49.79±12.27 to 4.46±5.24 in Subset B (p<0.05) (Figure 3).

Statistical comparison showed no significant differences between groups in pain (p=0.90) or stiffness (p=0.17) scores at three years, indicating comparable symptom relief. However, Subset A demonstrated significantly better scores in functional difficulty (p=0.037), with narrower confidence intervals, suggesting more consistent improvement in mobility-related tasks.

SF-36

Both Subset A and Subset B demonstrated significant improvements across all SF-36 domains from baseline to the three-year follow-up (p<0.001 for all comparisons). In Subset A, physical functioning improved from 11.79±15.13 to 71.53±19.97 (95% CI: 68.22-74.84), while Subset B showed an increase from 10.00±11.58 to 83.33±17.86 (95% CI: 76.46-90.20). Similarly, notable gains were observed in role limitations due to physical health, emotional well-being, pain, and general health domains in both groups (Table 5).

**Table 5:** Three-year outcomes of quality of life in both study groups (Subsets A and B) This table presents results from the SF-36 at three years, showing scores across multiple domains (physical functioning, role limitations, emotional well-being, pain, and general health). Data are reported as mean±standard deviation with 95% CI. Between-group comparisons were performed using independent t-tests, and p-values are reported. This allows the evaluation of whether inter-limb component size variation influenced broader patient-reported quality-of-life measures. SF-36: 36-Item Short-Form Health Survey

SF-36 questionnaire outcomes		Baseline	6 weeks	1 year	3 years	P-value (baseline vs. 3 years)	95% CI
Physical functioning	Subset A	11.79±15.13	56.12±22.94	74.60±17.64	71.53±19.97	<0.001	38.67 to 41.53
	Subset B	10.00±11.58	61.92±20.25	80.19±16.64	83.33±17.86	<0.001	37.73 to 45.27
Role limitations due to physical health	Subset A	6.25±23.25	74.82±41.58	95.77±16.26	86.01±22.63	<0.001	38.91 to 41.69
	Subset B	3.85±15.32	75.96±40.30	96.15±19.61	98.96±5.10	<0.001	38.73 to 46.07
Role limitations due to emotional problems	Subset A	27.14±43.17	77.78±39.94	95.83±17.42	88.31±20.54	<0.001	33.66 to 36.34
	Subset B	24.36±40.61	83.33±36.82	96.15±19.61	100.00±0.00	<0.001	34.31 to 41.09
Pain	Subset A	32.36±23.10	63.91±18.81	80.94±15.79	84.10±15.05	<0.001	36.38 to 39.42
	Subset B	27.40±23.22	69.23±17.89	83.37±11.44	84.58±15.44	<0.001	37.53 to 44.67
General health	Subset A	50.89±17.18	72.28±11.86	77.90±12.44	78.47±12.57	<0.001	35.20 to 37.80
	Subset B	49.81±20.76	73.27±10.19	75.96±9.59	69.17±10.90	<0.001	34.81 to 41.59
Health change	Subset A	24.46±19.54	79.71±14.34	92.46±12.66	90.30±17.04	<0.001	34.31 to 37.09
	Subset B	25.96±21.77	84.62±14.28	91.35±12.13	83.33±19.03	<0.001	33.51 to 40.49

Statistical comparison revealed that Subset B achieved significantly higher scores in physical functioning (p=0.005), role limitations due to physical health (p=0.004), and emotional problems (p=0.004) than Subset A, despite having a one-size difference in implant components between knees. Pain and general health scores were comparable between groups (p>0.05), indicating that component size asymmetry did not negatively impact perceived pain relief or overall health perception.

## Discussion

This prospective analysis provides statistically robust and clinically meaningful evidence on the impact of femoral and tibial component size variation in simultaneous bTKA. Our findings emphasize that prosthesis sizing, while anatomically individualized, plays a critical role in optimizing postoperative outcomes. The observed variability in femoral, tibial, and patellar sizes across the Indian cohort reinforces the need for personalized implant selection to accommodate native anatomical differences.

Both groups exceeded the MCID benchmarks for PROMs; the results should be interpreted in terms of practical relevance: Subset A patients reached slightly higher knee-specific scores (KSS), whereas Subset B patients reported better perceived physical functioning and emotional well-being. This observation may be explained by the fact that individualized sizing in Subset B allowed surgeons to match each knee more closely to its native morphology. Such personalized reconstruction can enhance proprioceptive comfort and reduce asymmetrical loading, leading to better subjective perception of physical functioning and emotional well-being, even when objective knee scores remain comparable. These findings highlight that modest asymmetry in component sizing does not translate into compromised patient recovery or satisfaction.

Importantly, no implant-related complications or revisions were observed (except for the isolated occurrence of a non-serious skin rash in Subset B that resolved without sequelae), and radiographs confirmed stable alignment across all cases, reinforcing the mid-term safety of individualized sizing. The consistent survivorship and functional recovery align with prior reports showing no adverse effect of size asymmetry in bTKA, while our study extends this evidence by incorporating quality-of-life measures in an Indian population.

In line with existing research [5,6,12,13], our study demonstrated a gradual and significant improvement in ROM, WOMAC scores, KSS, and SF-36 questionnaire in both cohorts, regardless of size variations. A retrospective study [6] of 324 bTKA patients, performed either on a single day or within 2-3 days simultaneously, revealed significant postoperative improvement in KSS and ROM, with no substantial differences between the two groups. Similarly, in another retrospective analysis of 268 simultaneous and staged bTKAs, no disparities were observed in cases with asymmetrical components concerning knee scores, pain, function, ROM, or complications [12]. Consistent with these outcomes, another retrospective assessment of anterior and posterior referenced bTKAs found no differences between symmetrical and asymmetrical components in terms of KSS and ROM [5]. Additionally, a retrospective analysis found no variance in outcomes between patients with symmetrical and asymmetrical femoral components [13]. While prior studies have focused primarily on KSS and ROM, our inclusion of SF-36 and WOMAC provides new insights into quality of life and pain-related outcomes in patients with component size asymmetry.

Discrepancies are reported in some studies regarding larger implant sizes [14-16]. Studies evaluating the effect of femoral component size reported that an increase in component size did not alter the anterior offset, overstuffing, or patellofemoral contact forces, parameters that can contribute to postoperative pain and restricted ROM after TKA [14,16]. The study also noted that tibial oversizing was associated with decreased ROM and functional scores (Knee Injury and Osteoarthritis Outcome Score (KOOS)), consistent with the findings of Bonnin et al. [15]. The findings from our study align more closely with these investigations, as, despite variations in both femoral and tibial component sizes, the outcomes were significant and similar to patients with same size implants. Notably, this study highlights the benefits of simultaneous bTKA with the study implants, with no revisions observed over three years in both cohorts, regardless of the component size used.

Our study found no substantial association between prosthesis size mismatch and postoperative complications. Patients in Subset B experienced lower rates of complications, including skin rash below the knee, while no complications related to the procedure or implant were noted in Subset A. These findings are consistent with prior research indicating that improper implant fit in bTKA leads to lower complication rates [17]. A meta-analysis revealed that simultaneous bTKA showed a lower risk of deep infection and respiratory complications but increased mortality, pulmonary embolism, and deep-vein thrombosis. No significant differences were observed in revision, superficial infection, arthrofibrosis, cardiac complications, neurological complications, and urinary complications between procedures [17]. Conversely, patients who received same-sized prostheses demonstrated better joint stability and alignment, thereby reducing the risk of complications.

Several publications have documented increased pain associated with mediolateral tibial overhang in both unilateral and bTKAs [15,18-21]. Oversizing the femur in the anteroposterior direction has been related to the cause of pain [22,23]. As no overhang was observed and patients reported a reduction in pain using the WOMAC and SF-36, it is safe to conclude that the implants utilized in this cohort of patients with varying femoral and tibial sizes can deliver the desired outcomes and improve the quality of life for TKA patients.

Finally, our findings underscore the importance of prosthesis sizing in predicting clinical outcomes in bTKA. Patients who had different-sized prostheses demonstrated comparable functionality, fewer postoperative problems, and better patient-reported outcomes similar to those who received same-sized prostheses. Orthopaedic surgeons should prioritize personalized prosthesis sizing and alignment to improve joint stability, biomechanics, and patient satisfaction. Collaborative efforts and ongoing research are required to establish uniform recommendations for prosthesis sizing in bTKA, which will raise the standard of care and advance the area of joint replacement surgery.

Limitations and future directions

The principal strength of this study lies in its prospective multicenter design, which enhances the external validity of findings and minimizes single-center or surgeon-specific bias. The study addressed a clinically pertinent and previously underexplored question, that is, whether inter-limb differences in component sizing during simultaneous bTKA affect outcomes, thereby contributing meaningful evidence to guide implant selection and surgical decision-making. Moreover, the standardized operative and postoperative protocols implemented across all participating centers, coupled with uniform data collection and periodic audits, ensured methodological consistency and reliable outcome assessment.

However, certain limitations must be acknowledged. Despite uniform training and protocol adherence, variations in surgical technique and surgeon experience across centers could introduce heterogeneity. The follow-up duration of three years provides valuable mid-term insight but does not capture potential long-term implant survivorship or late complications. Additionally, unmeasured patient-related confounders, including anatomical variations, preoperative activity levels, and comorbidities, may have influenced functional outcomes, although baseline characteristics were comparable between groups. Future large-scale studies with extended follow-up and multivariate adjustments for such confounders will be essential to validate these findings. Future research with longer-term follow-up, larger multicenter cohorts, and stratified analyses will be essential to confirm the durability of outcomes and inform evidence-based guidelines for prosthesis sizing strategies in bTKA.

## Conclusions

This prospective multicenter study provides evidence that individualized component sizing in simultaneous bTKA, including cases where a one-size difference exists between knees, does not adversely affect short- to mid-term outcomes. Both symmetric and asymmetric cohorts demonstrated significant improvements in ROM, knee-specific scores, and broader quality-of-life measures, with stable radiographic findings and no implant-related complications or revisions over three years.

A key strength of this study is its prospective design across multiple high-volume centers in India, allowing the systematic assessment of a relatively underexplored question in bTKA. Unlike prior retrospective series focusing primarily on knee scores and ROM, this study incorporated validated PROMs, including WOMAC and SF-36, to capture a broader perspective of patient recovery. The absence of revisions and the radiographic confirmation of well-aligned, stable implants further strengthen the robustness of the findings.

These results suggest that minor asymmetry in component selection, when guided by anatomical considerations, is a safe and clinically acceptable practice. The study adds novel mid-term data from an Indian population, where variability in native knee morphometry is pronounced, highlighting the relevance of individualized sizing strategies. Longer follow-up and larger cohorts will be important to validate these findings and to determine whether any long-term differences emerge between symmetric and asymmetric sizing approaches.
